# Psychomotor and Basic Cognitive Abilities in Professional Athletes: A Systematic Review

**DOI:** 10.5114/jhk/203566

**Published:** 2025-11-20

**Authors:** Izabela Huzarska-Rynasiewicz, Diogo V. Martinho, Adilson Marques, Marcelo de Maio Nascimento, Élvio Rúbio Gouveia, Andreas Ihle, Krzysztof Przednowek

**Affiliations:** 1 Faculty of Physical Culture Sciences, Medical College of Rzeszów University, University of Rzeszów, Rzeszów, Poland.; 2 Faculty of Sport Sciences and Physical Education, University of Coimbra, Coimbra, Portugal.; 3 LARSYS, Interactive Technologies Institute, Funchal, Portugal.; 4 CIPER, Faculty of Human Kinetics, University of Lisbon, Lisbon, Portugal.; 5 Núcleo de Investigación en Ciencias del Movimiento, Universidad Arturo Prat, Iquique, Chile.; 6 Department of Physical Education, Federal University of Vale do São Francisco, Petrolina, Brazil.; 7 Department of Physical Education and Sport, University of Madeira, Funchal, Portugal.; 8 Swiss Center of Expertise in Life Course Research LIVES, Geneva, Switzerland.; 9 Department of Psychology, University of Geneva, Geneva, Switzerland.; 10 Center for the Interdisciplinary Study of Gerontology and Vulnerability, University of Geneva, Geneva, Switzerland.

**Keywords:** athletes, cognitive abilities, psychomotor abilities

## Abstract

There are several ways to describe psychomotor and cognitive abilities in the context of sport performance, including psychomotor abilities, cognitive functioning, perceptual-cognitive skills, exercise-cognition, and motor-cognitive abilities. This review aimed to identify methods for measuring the aforementioned concepts within the context of relevant terminology. Studies examining psychomotor performance, as well as attentional, perceptual, and visual processes, were selected from three online databases: PubMed, Scopus, and Web of Science. Twenty-eight (28) studies were included in the review. The results were divided into sample characteristics and methodological details, including nomenclature specific to performance, methods, and outcomes. The studies were also categorised in the context of comparisons by the competitive level, sex and sport. Analysis showed that the most frequent basis of comparison included athletic performance. Computer-based methods occurred with the greatest frequency across all sport disciplines. Outcomes were typically reported in milliseconds, focusing on reaction time or accuracy. There was no consistency in the presentation of performance nomenclature and performance procedure. Addressing the selection and description of methods is relevant as it can contribute to a more effective research intervention design.

## Introduction

Participating in elite sports demands a high level of mental and physical attributes (Demulier et al., 2013). Within the psychological realm, cognitive and psychomotor abilities are crucial for performance of elite athletes (Kalén et al., 2021; Scharfen and Memmert, 2019; Skala and Zemkowa, 2022; Vestberg et al., 2012), as they significantly influence decision-making (Broadbent et al., 2015; Roca et al., 2013; Scharfen and Memmert, 2019). Examples of these essential abilities include visual search, situational information processing, discrimination of situational differences, and response types (Chainken et al., 2000). Although cognitive and psychomotor abilities are often treated as identical aspects within the psychological domain, they represent distinct concepts that warrant a comprehensive review.

Psychomotor ability refers to the precision and coordination of movement. It involves the selection and processing of information, allowing individuals to execute movements adequately (Kim et al., 2017). The result of this process is a movement response to visual or auditory signals (Paul et al., 2011), which is associated with simple motor activities (Nuri et al., 2013). In summary, psychomotor abilities are linked to executing movement with precision and coordination (Habay et al., 2021; Hindmarch, 2014). Cognitive abilities, on the other hand, pertain to the reception and interpretation of information within the mental domain (Kalén et al., 2021). These abilities can be categorized into two related groups: basic cognitive processes and higher cognitive functions (Butzbach et al., 2019; Paz-Alonso et al., 2013). Basic cognitive processes include processing speed, attention, and perception.

In contrast, higher cognitive functions encompass the interaction of multiple basic cognitive processes, such as working memory capacity and cognitive decision-making (Glisky, 2007). These functions involve tasks requiring participants to choose between different options, engage in judgment and decision-making, and anticipate outcomes (Kalén et al., 2021). While psychomotor abilities are primarily related to movement, cognitive functions are closely tied to mental operations and the foundations of the nervous system (Scharfen and Memmert, 2019). It is important to note that the terminology of these two concepts is often used interchangeably in sports science and psychology.

Researchers have defined cognitive and psychomotor abilities in various ways, such as perceptual-cognitive abilities (Williams and Ericsson, 2005), psychomotor abilities (Przednowek et al., 2019), cognitive-motor abilities (Wang et al., 2020), motor-cognitive abilities (Huzarska et al., 2023; Musculus and Raab, 2022), and sensory-cognitive abilities (Nuri et al., 2013). It is essential to analyse cognitive and psychomotor abilities as separate categories (Ree and Carretta, 1992), as they represent distinct concepts. The lack of consensus regarding terminology and assessment methods can significantly impact the interpretation of study conclusions (Voss et al., 2010). Previous reviews have examined the effects of mental fatigue on athletes (Dong et al., 2022; Habay et al., 2021; Skala and Zemkowa, 2022), but they often overlooked methodological differences among studies. Additionally, other reviews have focused exclusively on cognitive abilities (Furley et al., 2023; Heilmann et al., 2022; Kalén et al., 2021; Scharfen and Memmert, 2018) without considering psychomotor abilities. Given the conceptual and methodological differences between cognitive and psychomotor abilities, there is a need for a review that combines studies examining both domains. Furthermore, summarising the literature will provide a conceptual framework for understanding psychomotor and cognitive abilities in the context of sports, highlighting the methodological issues related to assessing both capacities.

This review aimed to summarise the research on psychomotor and cognitive performance in elite athletes, focusing on the methods, tools, and variables used for assessment. Concepts from both sports science and psychology were integrated by comparing psychomotor and cognitive performance. This distinction is important for addressing inconsistencies in the use of these concepts within the sports domain. By accurately defining and diagnosing these concepts, researchers can select and apply research methods more effectively. The specific aims of this review were as follows: (1) to verify the terms used to delineate psychomotor and cognitive abilities, ensuring a consistent framework for their application in research; (2) to focus on core functions, analysing which basic cognitive abilities or psychomotor skills are linked to perception, attention, or responding to a stimulus, while excluding broader constructs such as executive functions, decision-making, anticipation, or critical thinking; and (3) to optimize research approaches by distinguishing and recommending appropriate methods and tools for assessing cognitive and psychomotor performance in professional athletes.

## Methods

The review was performed in accordance with PRISMA Guidelines and Cochrane recommendations (Higgins et al., 2019; Page et al., 2021). The protocol was registered in the Open Science Framework on April 8, 2024.

### Eligibility Criteria

The inclusion criteria were guided by the Participants, Exposure, Comparators, Outcomes, and Study Design (PECOS) framework, as follows: (1) Participants: adult athletes (mean age > 18 years) classified as elite, professional or trained individuals; (2) Exposure: assessment of cognitive and psychomotor abilities, including attention and performance related to these abilities; (3) Comparator: comparison of athletes based on a competitive level, gender or against a control group; (4) Outcomes: measures of psychomotor and cognitive performance; (5) Study Design: observational studies. Studies that included Paralympic athletes were excluded as well as interventional studies that focused on training interventions. Only papers written in English and Polish were consulted and there were no defined restrictions regarding the year of publication or geographical location.

### Information Sources and Search Strategy

Three online databases were consulted: PubMed, Scopus and Web of Science. The search encompassed relevant publications available up to the 12^th^ of January, 2024. The search strategy used was: (“reaction time” OR “eye-hand coordination” OR “psychomotor performance” OR “psychomotor*” OR “psychomotor abilit*” OR “psychomotor skill*” OR “motor-cognitive” OR “cognitive abilit*” OR “cognitive performance” OR “perceptual-cognitive” OR “visuomotor” OR “visual skill*”) AND (athlet* OR sport*) AND (expert* OR athlet*) AND (adult).

### Selection Process

An automated procedure was executed using EndNote 20.6 for Windows (Clarivate) to prevent duplication of records. Manual screening was also carried out to ensure all duplicates were excluded. Two independent reviewers initially checked the titles and abstracts. Afterwards, full studies were screened following the eligibility criteria previously mentioned. When discrepancies occurred, a third external reviewer was consulted to guarantee agreement by consensus.

### Data Items, Extraction and Synthesis

The first author (I.H.-R.) conducted the data extraction process, collecting relevant information using a structured template. This comprehensive datasheet included all pertinent details and essential information. For each study, the following information was organized: (i) sample characteristics: (country, sample size, age, competitive level, training experience, sport); (ii) methodological details: study design, performance terminology and procedures, tools used, main outcomes and the respective unit of measurement; (iii) the key findings of each study.

The information from each paper was presented in tables and compiled in figures to highlight central points regarding sample characteristics, methodological issues, and results.

### Risk of Bias

The Quality Assessment Tool for Observational Cohort and Cross-Sectional Studies was used to assess the risk of bias for each study (National Heart, Lung, and Blood Institute, 2019). This tool includes fourteen questions related to various aspects of the research, such as the research question, study population, recruitment from the same population with uniform eligibility criteria, sample size, assessment of exposure before outcome measurement, an adequate timeframe to observe effects, different levels of the exposure of interest, exposure measures and assessment, repeated exposure, blinding, follow-up, and statistical analysis. In the present review, the following questions were not considered due to their inapplicability to the design of studies included in this review: question 8 (different levels of the exposure of interest), question 10 (repeated exposure assessment), question 12 (blinding of outcome assessors), and question 13 (follow-up). Two independent authors assessed the risk of bias; in the event of disagreement, a third experienced author made the final decision.

## Results

### Study Selection and Identification

**Figure 1:**
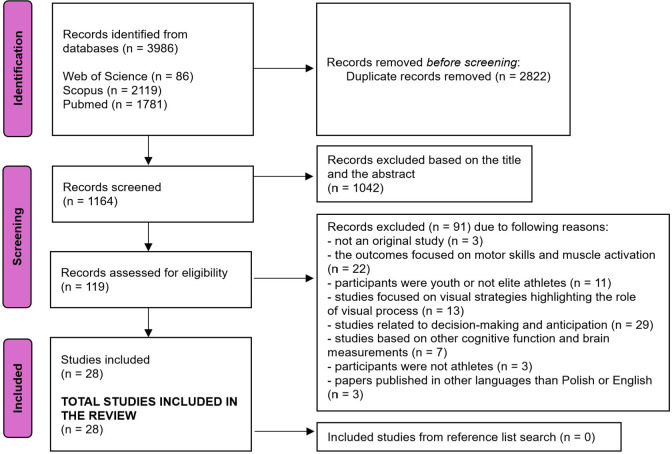
Flow chart of the study selection procedures.

The initial search across three databases resulted in the identification of 3,986 paper records. After removing 2,822 duplicates, 1,169 records were screened based on their titles and abstracts, leading to the deletion of 1,042 records. This process culminated in a full-text screening of 119 studies. Ninety-one (91) studies were excluded due to the following eight reasons: not an original study (n = 3), the outcomes focused on motor skills and muscle activation (n = 22), the sample included youth participants or not elite athletes (n = 11), visual strategies highlighting the role of the visual process were applied (n = 13), studies related to decision-making and anticipation (n = 29), brain measurement and cognitive functions were considered, which were not related to psychomotor or cognitive abilities (n = 7), study subjects were non-athletes (n = 3), papers published in other languages than Polish or English (n = 3). Finally, 28 studies met the eligibility criteria and were included in this review (Figure 1).

### Study Characteristics

The characteristics of the included studies are detailed in Tables 1 and 2. Among the considered studies, four were conducted in China (Chan et al., 2011; Cojocariu et al., 2019; Nian et al., 2023; Shao et al., 2020), while three were conducted in Poland (Markowski et al., 2023; Śliż et al., 2022, 2023). In total, the review encompassed 3,482 athletes, with sample sizes ranging from 12 (Oudejans et al., 1997) to 1,319 (Tønnessen et al., 2013).

**Table 1:** Characteristics of the participants examined in each study.

Study	Country	Sample size (N)		Age (yrs)	Competitive level	Training experience (yrs)	Sport discipline
Alves et al. (2013)	Brazil	154		24.85 ± 4.40 20.55 ± 1.23 17.58 ± 0.92 16.27 ± 1.06 23.33±3.04 21.55±1.50 17.33±1.13 16.45±1.53	Adult professional M Adult professional F Junior professional M Junior professional F Adult control M Adult control F Junior control M Junior control F	11.61 ± 4.75 9.66 ± 1.5 5.25 ± 2.43 5.43 ± 1.94	Volleyball
Bennett et al. (2020)	NR	110		19 -55 30.70±6.7	National	NR	Boxing, MMA, MA
Bianco et al. (2008)	Italy	60		24.1 ± 5.13 29.4 ± 4.19	Professional Amateur	3.8 ± 3.97 14.8 ± 5.16	Boxing
Chan et al. (2011)	China	60		20.63±2.11 20.63 ± 2.11	National	>5 non	Fencing
Cojocariu et al. (2019)	Spain	53		20.2 ±1.7 31.7 ±5.9	International University	>12 yrs NR	Qwan ki do
Cona (2015)	Italy	30		43±8.6	International	NR	Ultra-marathon run
Eriksson et al. (2023)	Sweden	93		18.21 ± 1.8 17.27 ± 1.05 17.50 ± 1.29	National National Control	7.62 ± 3.03 12.17 ± 2.79 non	Biathlon, alpine ski racing
Faro et al. (2020)	NR	34		26.5 ± 7.9 25.2 ± 5.8	Professional Amateur	>10 <1	Judo
Garrido-Palomino et al. (2020)	NR	35		34.7 ± 6.2	International	11.1 ± 7.0	Sports climbing
Harmenberg et al. (1991)	Sweden	14		23.1 19.5	International Beginners	11.7 1.25	Fencing
Hülsdünker et al. (2021)	Germany	19		21 ± 5	International	13 ± 5	Badminton
Kida et al. (2021)	Japan	193		23.4 ± 2.1 22.1 ± 1.9	International National Non-athletes	NR	Baseball, tennis
Liu et al. (2018)	Taiwan	159		21.57 ± 2.37 21.19 ± 1.57 21.09 ± 1.70 20.74 ± 1.74 20.23 ± 0.82 20.53 ± 0.68	National F National M Beginners Non-athletes	NR	Karate
López Del Amo et al. (2018)	NR		30	26 ± 2 28 ± 3 29 ± 3	International	NR	110 m hurdles
Lynch et al. (2019)	France		16	24.71 ± 2.43 27.56 ± 10.69	National Beginners	16.29 ± 4.23	Rugby
Markowski et al. (2023)	Poland		65	25.9 ± 7.6	National	NR	Speedway riders
Nian et al. (2023)	China		42	24.46 ± 1.43 22.33 ± 1.05 21.38 ± 1.43	National University Beginners	NR	Basketball
Oudejans et al. (1997)	United States		12	24 ± 22–31	International Beginners	15 ±	Basketball
Petrenko et al. (2021)	Russia		38	NR	National	NR	Track and field
Russo et al. (2022)	NR		95	28.62 ± 8.39 27.30 ± 7.49 29.97 ± 9.73 29.04 ± 8.25	NR	NR 14.73 ± 7.78 8.44 ± 7.82	Open skill Closed skill
Shao et al. (2020)	China		32	29.36 ± 0.71 17.89 ± 1.15 23.50 ± 0.34	International Beginners Non-athletes	18.13 ± 2.48 2.84 ± 0.57	Shooting
Spierer et al. (2010)	United States		35	20.7 ± 2.3	National	NR	Soccer, lacrosse
Śliż et al. (2023)	Poland		118	19.60 ± 3.16	National	7.69 ± 2.43	Handball
Śliż et al. (2022)	Poland		49	26.5 ± 5.2	National	NR	Rugby
Tønnessen et al. (2013)	NR		1319	16–47	International	NR	Sprint
Van Leeuwen et al. (2017)	Netherlands		17	19.9 ± 1.8 21.6 ± 1.7	International Non-racing	8.4 ± 3.0	Racing
Xie et al. (2022)	China		527	NR	National	NR	Track and field
Yildiz et al. (2020)	Turkey		73	22.0 ± 2.7	National	10.7 ± 2.9	Soccer

NR (Not Reported); yrs (years); Levels: International, National/Professional, University/College, Beginners/Amateur, Non-athletes/Control; MA (martial arts), MMA (mixed martial arts)

**Table 2:** Summary of methodological details of each psychomotor and basic cognitive abilities studies.

Study	Study design		Methodological details									
			Performance nomenclature		Performance procedures		Tools		Outcome variables		Outcome units	
Alves et al. (2013)	Cross-sectional		Perceptual-cognitive expertise		Computer tests		Task Switching, Useful Field of View, Visual short-term memory, Stopping, Flanker, Change detection		Mean RT, Mean accuracy		Miliseconds, Seconds	
Bennett et al. (2020)	Cross-sectional		Cognitive performance		CNS Vital Signs program Trails A via the iCOMET		Symbol digit, Coding, Finger-tapping, Stroop-like test		Correct answers, Reaction time		Numbers, Miliseconds	
Bianco et al. (2008)	Cross-sectional		Cognitive functions		CogSport computerized NP test battery		Detection task, Identification task, Monitoring task		Mean reaction time, Errors, Reaction time change		Miliseconds, %, %	
Chan et al. (2011)	Cross-sectional		Cognitive capability, Executive functions		Computer test		Go/no-go task		Simple RT mean, Mean commission error, Mean omission error		Miliseconds	
Cojocariu et al. (2019)	Cross-sectional		Visual choice reaction time		Computer tests		Visual choice, reaction time		Mean reaction time		Miliseconds	
Cona (2015)	Cross-sectional		Cognitive functioning		Computer tests		Inhibitory control task		Mean accuracy, Mean reaction time		Number Miliseconds	
Eriksson et al. (2023)	Cross-sectional		response inhibition		Computer test		Stop-signal task		Reaction time go trials, Signal-respond reaction time, Stop-signal reaction time, Stop-signal delay, Accuracy		Miliseconds Miliseconds %	
Faro et al. (2020)	Cross-sectional		Cognitive performance		Computer tests, E-prime 2.0		Go/NoGo, Stroop color-word, Matching test		Accuracy, Response Time, Response Time variability		%, Miliseconds, Miliseconds	
Garrido-Palomino et al. (2020)	Cross-sectional		Attention		Vienna Test System 26.04		Signal detection, Determination task		Visual scanning accuracy, Selective attention accuracy, Speed response		% % Miliseconds	
Harmenberg et al. (1991)		Cross-sectional		Fencing Performance		Self-design protocol		Hitting target		Reaction time, Movement time, Total time	Miliseconds, Miliseconds, Miliseconds
Hülsdünker et al. (2021)		Nonrandomized controlled trials		Reaction speed		Lab Test		Reaction task		Reaction time, Monosensory reaction time, Multisensory reaction time	Miliseconds, Miliseconds, Miliseconds
Kida et al. (2021)		Cross-sectional		Go/Nogo reaction, simple reaction time		Computer tests		Simple reaction task Co/No Go task		Simple reaction time, Go/No Go reaction time, Commission error rate, Go/No Go, Error rate	Miliseconds, Miliseconds % %
Liu et al. (2018)		Cross-sectional		Simple and choice reaction time		FITLIGHT Trainer™ System		Simple reaction time, Choice reaction time		Response time	Miliseconds
López Del Amo et al. (2018)		Cross-sectional		Reaction time		provided by the IAAF		Reaction time		Reaction time	Seconds
Lynch et al. (2019)		Cross-sectional		Perception-action performance		Computer Assisted Virtual Environment		Perception task, Perception-action task		Correct response, Response time	% Seconds
Markowski et al. (2023)		Cohort study		Response time		Pegasus Speedway telemetry system		Real-time analysis		Reaction time	Seconds
Nian et al. (2023)		Cross-sectional		Visual search response		E-prime 3.0		Visual search task		Total reaction time, Correct rate	Miliseconds, %
Oudejans et al. (1997)		Cross-sectional		Movement initiation		Self-design protocol		Perceptual location task		Mean movement initiation time, Incorrect responses	Miliseconds
Petrenko et al. (2021)		Cross-sectional		Psychomotor characteristics		Psychophysio- logical Test System		Real-time analysis		Simple visual-motor response, Simple sensorimotor response, Sensorimotor dynamic coordination, Dynamic visual, response speed	Miliseconds, Miliseconds, Ratio, Number
Russo et al. (2022)		Cross-sectional		Visual search ability		Psychtoolkit		Visual search test		Reaction Time, Correct responses	Miliseconds
Shao et al. (2020)		Cross-sectional		Executive functions		E-prime		Flanker task		Accuracy, Reaction time	Miliseconds
Spierer et al. (2010)		Cross-sectional		Response to stimuli		Cybex Reactor		Cybex reactor		Reaction time, Move time	Seconds, Seconds
Śliż et al. (2023)		Cross-sectional		Psychomotor abilities		Test2Drive Computer System		SIRT, CHORT, HECOR, SPANT		Reaction time, Movement time, Correct answers	Miliseconds, Miliseconds %
Śliż et al. (2022)		Cross-sectional		Psychomotor abilities		Test2Drive Computer System		SIRT, CHORT, HECOR, SPANT		Reaction time, Movement time, Correct answers	Miliseconds, Miliseconds %
Tønnessen et al. (2013)		Cross-sectional		Reaction Time		IAAF official website		Reaction time		Mean Reaction time	Seconds
Van Leeuwen et al. (2017)		Cross-sectional		Reaction time and visual-motor performance		Tatuus Formula Renault 2.0 chassis		Choice reaction Time task, Visual-motor task		Choice reaction time, Root mean square	Miliseconds
Xie et al. (2022)		Cross-sectional		Reaction Speed		Swiss OF02-ATΩ starting foul monitor to monitor		NR		Average starting, Reaction time	Seconds
Yildiz et al. (2020)		Cross-sectional		Reaction Time		Lafayette MOART system		Visual reaction Test		Reaction time	Miliseconds

NR (Not Reported); RT (reaction time); s (seconds); ms (milliseconds); % (percent of correctness/accuracy); IAAF (International Association of Athletics Federation International); SIRT (Simple Time RT); CHORT (Choice RT); HECOR (Hand-eye coordination); SPANT (Spatial anticipation)

Of note, eight studies explored psychomotor and cognitive variables in team sports athletes, accounting for 25% of the total (Alves et al., 2013; Kida et al., 2005; Lynch et al., 2019; Nian et al., 2023; Spierer et al., 2010; Śliż et al., 2022, 2023; Yildiz et al., 2020). Furthermore, more than 60% of the studies focused on individual sports. Seven studies examined psychomotor and cognitive abilities specifically in combat sports (Bennett et al., 2020; Bianco et al., 2008; Chan et al., 2011; Cojocariu et al., 2019; Faro et al., 2020; Harmenberg et al., 1991; Liu et al., 2018), while another five focused on track and field disciplines (Cona et al., 2015; López Del Amo et al., 2018; Petrenko et al., 2021; Tønnessen et al., 2013; Xie et al., 2022). Other sports, including speedway, car racing, climbing, shooting, badminton, biathlon, and skiing, were also investigated, although each of these sport disciplines was considered in only one study. Additionally, one study categorised activities into open and closed sports (Russo et al., 2022), thereby combining various sports in their analysis.

**Figure 2:**
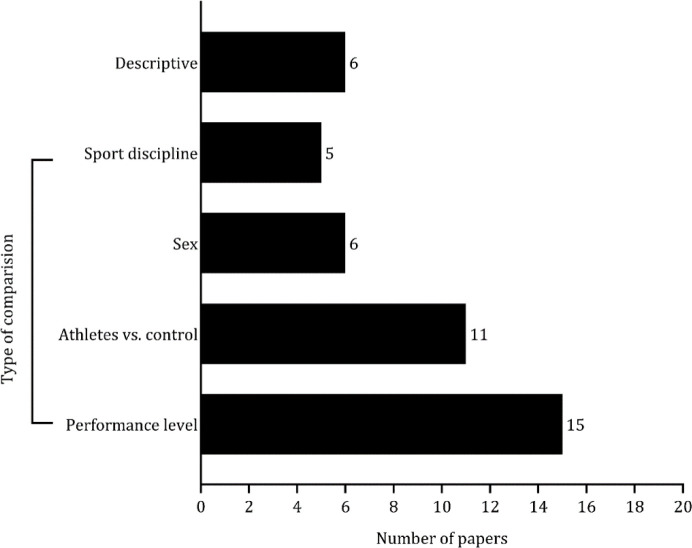
Results of the studies based on the type of comparisons.

The studies were organized based on the type of comparisons made. As shown in Figure 2, 12 studies contrasted athletes with different competitive or skill levels (Alves et al., 2013; Bianco et al., 2008; Chan et al., 2011; Cona et al., 2015; Harmenberg et al., 1991; Kida et al., 2005; Liu et al., 2018; Lynch et al., 2019; Nian et al., 2023; Oudejans et al., 1997; Shao et al., 2020; Śliż et al., 2022). Eight studies assessed differences in psychomotor and cognitive abilities between professional athletes and a control group (i.e., the general population) (Alves et al., 2013; Chan et al., 2011; Cojocariu et al., 2019; Kida et al., 2005; Liu et al., 2018; Shao et al., 2020; Śliż et al., 2023; Van Leeuwen et al., 2017). Comparisons based on sex were performed in five studies (Bennett et al., 2020; Eriksson et al., 2023; Spierer et al., 2010; Tønnessen et al., 2013; Xie et al., 2022). Lastly, four studies examined differences in psychomotor and cognitive outcomes by the type of sport (Eriksson et al., 2023; Kida et al., 2005; Petrenko et al., 2021; Russo et al., 2022), while five studies solely described the levels of these outcomes (Garrido-Palomino et al., 2020; Harmenberg et al., 1991; Hülsdünker et al., 2021; Markowski et al., 2023; Yildiz et al., 2020).

### Methodological Issues and Outcomes

**Figure 3:**
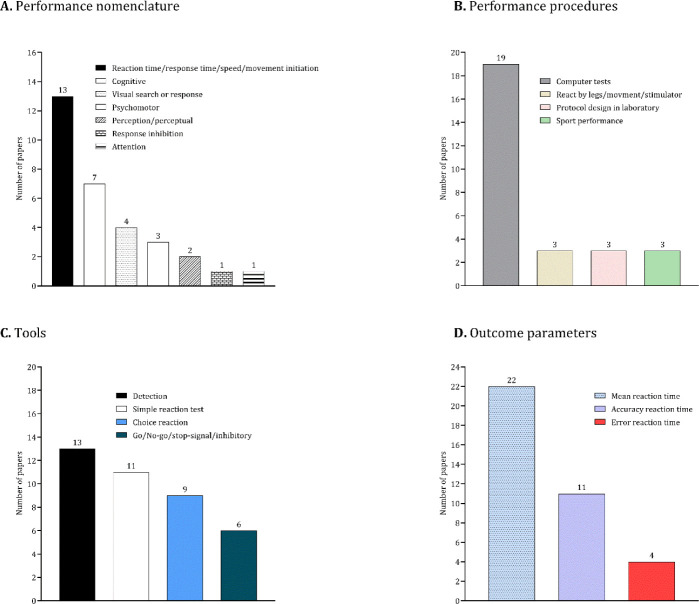
Results of the studies based on the performance nomenclature (A), performance procedures (B), tools (C) and outcome variables (D).

Table 2 summarizes the methodological details of each study. As depicted in Figure 3, 13 studies (approximately 42%) utilized the performance nomenclature “reaction time/response time/speed/movement initiation”, while seven studies (approximately 31%) focused on “cognitive” performance. Other performance terminologies included “psychomotor”, “perception/perceptual”, “visual search/response”, “response inhibition”, and “attention”. In terms of performance procedures, 19 studies (approximately 39%) employed a computer system. The most common tests used to extract outcomes were detection (13 studies), the simple reaction time test (11 studies), and the choice reaction test (9 studies). Notably, the most prevalent outcome examined was the mean value of reaction time, assessed in 22 studies.

### Comparisons by Competitive Level, Sex and Sport

Tables 3–5 present comparisons of psychomotor and cognitive abilities across competitive levels, sex, and types of sport. In 56 comparisons, elite or expert athletes generally demonstrated better performance than those at lower competitive levels or control groups. Conversely, novices, non-experts or debuting athletes exhibited superior performance outcomes in 12 of the 52 comparisons (approximately 23%). Regarding sex comparisons, the findings across studies were inconsistent, with two studies favouring males and two favouring females. Additionally, only three studies examined psychomotor and cognitive abilities by the type of sport, and no significant differences were found.

**Table 3:** Comparisons of athletic participation among participants of various sports levels and a control group.

COMPARISION BY THE SPORTS LEVEL				
Study	Outcome	Groups: mean ± SD	*p-value*	Favours*
Alves et al. (2013)	Single switching task RT	Elite: 570.88 ± 8.42 Control: 596.18 ± 9.30	0.05	Elite
	Go task RT	Elite: 746.48 ± 15.13 Control: 656.66 ± 16.45	0.001	Elite
	Change detection task	Elite: 7.23 ± 0.17 Control: 7.70 ± 0.17	0.006	Elite
Bianco et al. (2008)	Detection task SRT 1^st^ trial	Elite: 0.244 ± 0.007 Debuting: 0.249 ± 0.007	0.005	Debuting
	Detection task SRT 2^nd^ trial	Elite: 0.247 ± 0.007 Debuting: 0.251 ± 0.008	0.028	Debuting
Chan et al. (2011)	Simple RT	Elite: NR Non-athletes: NR	0.20	No differences
	Motor speed	Elite: NR Non-athletes: NR	0.84	No differences
	Go/no go	Elite: NR Non-athletes: NR	0.94	No differences
Cojocariu et al. (2019)	VCTR 1	Elite: NR; Non-athletes: NR	0.018	Elite
	VCTR 1 Red dots, white background DH	Elite 364.4 ± 12.3 Non-athletes 408.02 ± 7.4	0.033	Elite
	VCTR 1 Red dots, white background NDH	Elite 361.8 ± 7.5 Non-athletes 404.3 ± 6.8	0.021	Elite
	VCRT 2	Elite: NR; Non-athletes: NR	0.005	Elite
	VCRT 2 Blue dots, white background DH	Elite 363 ± 11.5 Non-athletes 405.3 ± 6.3	0.015	Elite
	VCRT 2 Blue dots, white background NDH	Elite NDH: 353 ± 8.5 Non-athletes 403.1 ± 6.3	0.004	Elite
	VCRT 3	Elite: NR; Non-athletes: NR	0.05	Elite
	VCRT 3 White dots, red background DH	Elite 367.7 ±15.6 Non-athletes 407.1 ± 7.3	0.52	Elite
	VCRT 3 White dots, red background NDH	Elite 368.4 ± 14.6 Non-athletes 405.9 ± 6.9	0.05	Elite
	VCRT 4	Elite: NR; Non-athletes: NR	0.023	Elite
	VCRT 4 Blue dots, red background DH	Elite 368 ± 13.5 Non-athletes 415.04 ± 6.5	0.01	Elite
	VCRT 4 Blue dots, red background NDH	Elite 382.5 ±9.4 Non-athletes 412.7 ± 6.7	0.096	Elite
	VCRT 5	Elite: NR ; Non-athletes: NR	0.043	Elite
	VCRT 5 White dots, blue background DH	Elite 367.5 ± 14.06 Non-athletes 405.2 ± 6.4	0.037	Elite
	VCRT 5 White dots, blue background NDH	Elite 375.8 ± 10.03 Non-athletes 408.5 ± 7.3	0.099	Elite
	VCRT 6	Elite: NR; Non-athletes: NR	0.009	Elite
	VCRT 6 Red dots, blue background DH	Elite 369 ± 13.5 Non-athletes 415.06 ± 7.1	0.02	Elite
	VCRT 6 Red dots, blue background NDH	Elite 360.8 ± 8.6 Non-athletes 406.3 ± 6.5	0.01	Elite
Cona (2015)	RT	Elite: 455 ± 16.97 Intermediate: 438 ± 8.90	0.01	Intermediate
Harmenberg et al. (1991)	Total Time (RT + MT)	Elite 0.607 ± 0.079 Non-athletes 0.731 ± 0.04	0.006	Elite
Kida et al. (2021)	Go/no go RT	Elite baseball: 293 ± 37 Intermediate Tennis: 332 ± 34	0.05	Elite
	Go/no go RT	Elite baseball: 293 ± 37 Non-athletes: 347 ± 46	0.01	Elite
Liu et al. (2018)	SRT	Elite: 292.33 ± 45.4 Novice: 306.33 ± 47.05 Non-athletes: 335.43 ± 73.05	0.001	Elite
	CRT	Elite: 352.11 ± 35.9 Novice: 376.28 ± 61.38 Non-athletes: 423.7 ± 63.58	0.001	Elite
Lynch et al. (2018)	Perception accuracy	Experts: 75.30 ± 31.92% Novices: 61.27 ± 38.13%	0.001	Novices
Nian et al. (2023)	Visual Search Reaction Time	Elite: 1388.82 ± 165.27 Novices: 1744.18 ± 213.58 Semi-elite: 1625.75 ± 197.31	0.001	Elite
	Initiation Reaction Time	Elite: 279.95 ± 18.31 Novices: 312.75 ± 29.58 Semi-elite: 304.64 ± 29.12	0.001	Elite
Oudejans et al. (1997)	Foot Movement Initiation Time	Experts: 350 Non-experts: 265	0.06	Non-experts
Shao et al. (2020)	RT Inconsistent Trails 300 IS	Experts: 183.05 ± 15.85 Controls: 210.70 ± 25.93	0.041	Experts
	RT Inconsistent Trails 300 IS	Novices: 175.82 ± 31.65 Controls: 210.70 ± 25.93	0.037	Novices
	RT Inconsistent Trails 400 ISI	Experts: 192.28 ± 20.63 Controls: 239.78 ± 43.29	0.041	Experts
	RT Inconsistent Trails 400 ISI	Novices: 195.15 ± 25.68 Controls: 239.78 ± 43.29	0.037	Novices
	RT Consistent Trails 300 ISI	Experts: 173.39 ± 16.37 Controls: 206.74 ± 36.18	0.015	Experts
	RT Consistent Trails 300 ISI	Novices: 165.50 ± 11.97 Controls: 206.74 ± 36.18	0.010	Novices
	RT Consistent Trails 400 ISI	Experts: 172.97 ± 20.45 Controls 227.92 ± 49.46	0.015	Experts
	RT Time Consistent Trails 400 ISI	Novices: 169.05 ± 19.38 Controls 227.92 ± 49.46	0.010	Novices
Śliż et al. (2023)	Simple RT	Elite rugby: 356.0 ± 34.4 Non-athletes: 332.4 ± 41.7	0.0389	Non-athletes
	Simple MT	Elite rugby: 158.1 ± 38.0 Non-athletes: 192.1 ± 47.4	0.0089	Elite
	Choice MT	Elite rugby: 170.3 ± 42.4 Non-athletes: 214.7 ± 48.0	0.0014	Elite
	Hand-eye Coordination MT	Elite rugby: 206.3 ± 31.4 Non-athletes: 243.0 ± 50.9	0.0065	Elite
Śliż et al. (2022)	Simple MT	Elite: 216.29 ± 32.71 Professionals: 201.54 ± 37.40 Intermediate: 185.32 ± 34.23	0.017	Intermediate
	Choice RT	Elite: 650.82 ± 45.07 Professionals: 670.17 ± 70.85 Intermediate: 698.71 ± 58.77	0.014	Elite
	Hand-eye Coordination MT	Elite: 259.71 ± 27.95 Professionals: 241.73 ± 39.15 Intermediate: 229.87 ± 31.52	0.017	Intermediate
Van Leeuwen et al. (2017)	RT	Elite: 431.6 ± 35.8 Non-athletes: 439.5 ± 34.2	0.6689	No significant differences

RT (reaction time); SRT (simple reaction time); NR (not reported); VCRT (visual choice reaction time); DH (dominant hand); NDH (non-dominant hand); * Favours meaning better performance

RT (reaction time); SRT (simple reaction time); NR (not reported); SD (standard deviation); MT (movement time); * Favours meaning better performance

**Table 4:** Comparisons of psychomotor/cognitive abilities by sex.

COMPARISION BY SEX				
Study	Outcome	Groups: mean ± SD	*p*-value	Favours*
Bennett et al. (2020)	Performance of psychomotor speed measure	Males: NR Females: NR	0.005	Females
Eriksson et al. (2023)	SSRT	Males: 136.78 ± 48.90 Females: 157.76 ± 34.98	0.018	Males
Garrido-Palomino et al. (2020)	RT	Males: 660 ± 46.8 Females: 679 ± 33.5	*p* > 0.05	No differences
Spierer et al. (2010)	RT	Males 0.1782 ± 0.189 Females 0.1317 ± 0.192	0.05	Females
Tønnessen et al. (2013)	RT	Males: 0.166 ± 0.030 Females: 0.176 ± 0.034	0.05	Males

RT (reaction time); SD (standard deviation); SSRT (stop-signal reaction time); NR (not reported); * Favours meaning better performance

**Table 5:** Comparisons of psychomotor/cognitive abilities by type of sport.

COMPARISION BY THE SPORT DISCIPLINE				
Study	Outcome	Groups: mean ± SD	*p-value*	Favours*
Eriksson et al. (2023)	SSRT	Alpine skiers 145.63 ± 40.93 Biathletes 150.47 ± 39.16 Non-athletes 149.99 ± 47.67	0.89	No differences
Kida et al. (2021)	Go/no go reaction time	Baseball: 293 ± 37 Tennis: 332 ± 34	0.05	Elite
Russo et al. (2022)	RT	OSA: 1023.6 ± 22.02 CSA: 1162.6 ± 28.1	0.94	OSA

RT (reaction time); SSRT (stop-signal reaction time); SD (standard deviation); OSA (open skill athletes); CSA (close skill athletes) * Favours meaning better performance

### Risk of Bias Assessment

Table 6 summarizes the risk of bias assessment for each study. The research objectives were clearly defined in 22 studies, accounting for approximately 78% of the total. Thirteen studies lacked a comprehensive description of the characteristics of the examined population. Other significant issues identified in the risk of bias assessment included the calculation of sampling power, which was conducted in only five studies, and the adjustment for confounding variables, which was addressed in only 25% of the studies. In contrast, the descriptions of dependent and independent variables were sufficiently provided in more than 90% of the studies.

**Table 6:** Risk of bias assessment using the Quality Assessment Tool for Observational Cohort and Cross-Sectional Studies.

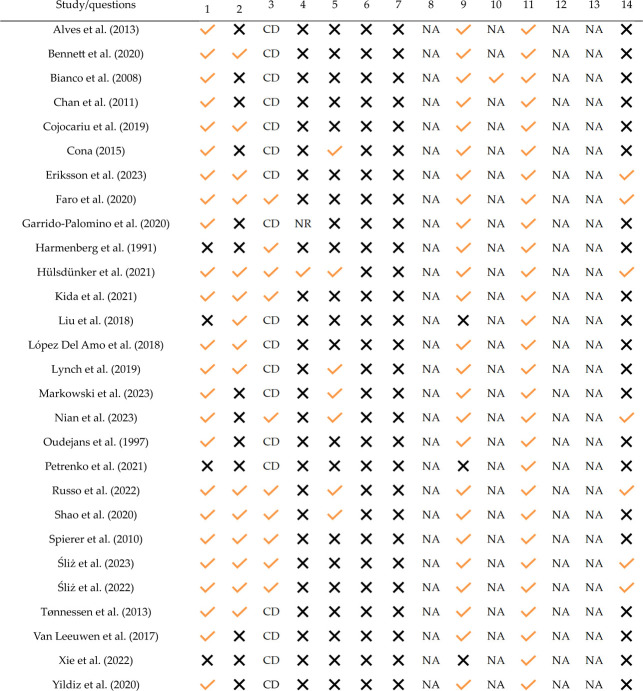

CD (cannot determinte); NA (not applicable)

## Discussion

The topic of cognitive abilities and psychomotor skills is frequently explored in research on professional sport (Habay et al., 2021; Kalén et al., 2019; Scharfen and Memmert, 2019). This review critically examines methodological details such as performance nomenclature as well as procedures and tools related to the assessment of psychomotor and cognitive abilities. The objective was to examine cognitive processes associated with perception, attention, and response to stimuli, with a particular emphasis on their measurement and analysis in scientific research. A notable challenge revealed by the review is the terminological inconsistency in describing cognitive and psychomotor measurements. The terms “reaction” or “response”, “perceptual/visual”, “psychomotor” and “cognitive processes” are often used interchangeably, even when referring to similar constructs and procedures. No specific rule was identified that characterized the method of use of these terms. The primary feature of this measurement was that it combined the fields of knowledge related to psychology, neuropsychology, sports training, and motor control (Block et al., 2017; Markov and Lebedinsky, 2014). For this reason, addressing the selection and description of methods is crucial (Mann et al., 2007), as it can contribute to the design of a more effective research intervention. The most frequently applied approach was computer-based. The methods utilized were generally broad rather than sport-specific, contrary to those which accounted for the contextual factors of athletic performance. The most measured outcomes included median reaction time, motor time or performance accuracy. Using such methods implies a risk that they may not fully reflect the cognitive processing of information under real-world conditions.

In the present review, we demonstrated significant variability in sample sizes across studies as study groups ranged from small to very large (Oudejans et al., 1997; Tønnessen et al., 2013). This may have introduced uncertainty in the selection of methodological approaches and the interpretation of results. The risk of bias analysis revealed that only a limited number of studies applied methods of justification to the sampling size selection.

Moreover, comparative analyses were conducted based on the study selection. These comparisons primarily concerned the sports level, as well as sex and the specific sport discipline. For the most part, better performance was observed in elite athletes. Surprisingly, there were 12 studies favoring novices, non-expert or debuting athletes when compared to the elite. In the expert-novice research paradigm, differences between skilled and less skilled athletes are evident due to greater experience in anticipation and direct perception (Jacobson and Matthaeus, 2014). The outcomes can differentiate among athletes in the context of a sport discipline thus appropriate methods should be adapted to the characteristics of the sport discipline (Kaluga et al., 2020). When representatives of different sports perform various tasks, comparing and making general conclusions about the impact of a sport discipline may be limited (Рiatysotska et al., 2023). In addition, there have been studies making comparisons with non-athletes. It is also well-known in the literature that sports training stimulates the neural transmission by enhancing the functioning of perception, attention and reaction, thus comparing athletes to non-athletes results in a preconceived advantage for athletes (Fadde, 2009; Sagdilek and Sahin, 2015; Suppiah et al., 2016).

The topic of psychomotor assessment has become multidisciplinary, and there is still a lack of answers regarding a human’s response and behaviours under situational and contextual conditions, as presented solely by computer-based measurements (Krivokapic and Tanase, 2016). Regarding the methodology details, computer-based methods were most frequent in the included studies. However, although the applied methods were generally called “computer methods”, no specific names of the employed system were provided. Such studies included only a description of the specific task or test, along with the proper way to perform it, yet no further specific details were provided.

When using computer-based methods, it may be difficult to find differences between athletes regardless of the type of sport they practise (Moşoi and Balint, 2015). Also, it is easier to understand and interpret the obtained results when they concern a real-situation task related to a perceptual-cognitive response (Hinz et al., 2021). For being effective in sport, the processing context and consequences of the made choice also matter (Voss et al., 2010) and in computer-based tests that part cannot be appropriately highlighted. Different sports require tasks of varying complexity levels and information to process; thus, comparisons between sports considering their cognitive dimensions are often fraught with the risk of error or inadequate representation of the subject (Aslan, 2018). Cognitive processing and its difficulty in a single or a complex task is closely related to tactical objectives in particular sport disciplines (Moreira et al., 2025). Moreover, these abilities can also depend on parental attitudes or personality traits, such as mental toughness (Vega-Díaz and González-García, 2025); thus, the topic is complex and requires a holistic approach.

The studies included in the present review were not entirely free from limitations. It needs to be highlighted that several critical gaps remain in the current body of research. First, the lack of standardisation in methodologies and nomenclature necessitates a unified framework to ensure consistency across studies. In this paper, we based our analysis on the nomenclature and findings reported by authors in their studies. Due to the critical approach, we attempted to identify potential differences in nomenclature and methods. To ensure accuracy, we conducted a risk of bias analysis. The primary limitation of the analysed studies was the lack of detailed description and a clear rationale for the selection of the study sample, which consequently influenced the methodology employed. Additionally, many studies failed to control for confounding variables, such as the duration of a simple reaction, when assessing a complex reaction. However, it is essential to note that, in most cases, both the dependent and independent variables, as well as the purpose of measurement, were described fairly and accurately, thereby contributing to the overall transparency of the analysis. The included studies were only in English, thus perhaps there exist more similar studies in other languages at a local research environment. Perhaps there are published papers that measure psychomotor or basic cognitive abilities but have been mistakenly called executive functions or otherwise. In the current work, no studies were found that included interventions or training effects on psychomotor or cognitive functioning. Furthermore, different sample sizes were considered; therefore, the obtained results may not be directly comparable. The key is to carefully select the experimental group and compare it with a suitable control group (Horoszkiewicz, 2024). However, in some of the studies, groups differed significantly in size and the skill level. Moreover, one can see clear predominance of male subjects. Next, the influence of contextual factors, such as environmental and discipline-specific factors, as well as the type of sport discipline, should be more in-depth and incorporated into future assessments. In the current review, both team and individual sport disciplines, along with Olympic and non-Olympic disciplines, were included, which implied a different choice of measurement methods, but not necessarily the naming of variables.

## Conclusions

The considered topic is important for understanding the differences and processes underlying the concepts of psychomotor and cognitive abilities in the context of sports performance. By addressing these challenges, future research can enhance the validity and applicability of its findings, thereby optimising evidence-based research procedures. This knowledge can be applied in learning, cognition, sports training, and practical tips for athletes. The correct diagnosis can help predict the athlete’s development and a situational disposition under training conditions (Szwarc et al., 2021). However, one may observe a lack of clear division in research between the presented abilities as well as the gold standard in such measurements. There is a great need to plan the research protocol and distinguish between the particular concepts with more precision. This review presents a valuable attempt to clarify these two topics and systematise measurement methods for psychomotor abilities and basic cognitive processes. It is a significant contribution to knowledge regarding future publications in sports science, psychology, and cognition in sports.
